# Stage Dependent Aberrant Regulation of Cytokine-STAT Signaling in Murine Systemic Lupus Erythematosus

**DOI:** 10.1371/journal.pone.0006756

**Published:** 2009-08-25

**Authors:** Matthew B. Hale, Peter O. Krutzik, Shamsher S. Samra, Janelle M. Crane, Garry P. Nolan

**Affiliations:** The Baxter Laboratory of Genetic Pharmacology, Department of Microbiology and Immunology, Stanford University School of Medicine, Stanford, California, United States of America; New York University School of Medicine, United States of America

## Abstract

Systemic lupus erythematosus (SLE) is a complex autoimmune disease of unknown etiology that involves multiple interacting cell types driven by numerous cytokines and autoimmune epitopes. Although the initiating events leading to SLE pathology are not understood, there is a growing realization that dysregulated cytokine action on immune cells plays an important role in promoting the inflammatory autoimmune state. We applied phospho-specific flow cytometry to characterize the extent to which regulation of cytokine signal transduction through the STAT family of transcription factors is disturbed during the progression of SLE. Using a panel of 10 cytokines thought to have causal roles in the disease, we measured signaling responses at the single-cell level in five immune cell types from the MRL*lpr* murine model. This generated a highly multiplexed view of how cytokine stimuli are processed by intracellular signaling networks in adaptive and innate immune cells during different stages of SLE pathogenesis. We report that robust changes in cytokine signal transduction occur during the progression of SLE in multiple immune cell subtypes including increased T cell responsiveness to IL-10 and ablation of Stat1 responses to IFNα, IFNγ, IL-6, and IL-21, Stat3 responses to IL-6, Stat5 responses to IL-15, and Stat6 responses to IL-4. We found increased intracellular expression of Suppressor of Cytokine Signaling 1 protein correlated with negative regulation of Stat1 responses to inflammatory cytokines. The results provide evidence of negative feedback regulation opposing inflammatory cytokines that have self-sustaining activities and suggest a cytokine-driven oscillator circuit may drive the periodic disease activity observed in many SLE patients.

## Introduction

Systemic Lupus Erythematosus (SLE) is a debilitating autoimmune disease that can damage multiple organs, induce chronic renal failure, and lead to severe morbidity and mortality. Current treatment regimens are limited to non-specific immune suppression and management of inflammatory symptoms. A characteristic feature of SLE is the presence of anti-nuclear autoantibodies (ANA) that form immune complexes with cellular debris and cause end-organ damage. Although the initiating events leading to SLE pathology are not understood, it is accepted that cytokine release from dysregulated immune cells plays a central role in promoting the autoimmune inflammatory state [1]–[9].

One of the key unanswered questions is how cytokines modulate the immune system and subvert its ability to control the autoimmune state — in particular, how intracellular signaling networks are “re-wired” by tonic stimulation with extracellular ligands. We hypothesized that abnormal regulation of cytokine signal transduction in immune cells is an integral feature of SLE and that changes in cytokine signaling would parallel disease progression and severity. Most cytokines, including those associated with SLE, signal through the Signal Transduction and Activators of Transcription (STAT) family of transcription factors. STAT proteins, upon being phosphorylated at activating residues, translocate from the plasma membrane to the nucleus where they drive numerous gene programs critical to immune function [10]. To quantify STAT signaling responses to cytokines during different phases of SLE we applied phospho-specific flow cytometry, an approach uniquely capable of measuring the biochemical activation of multiple pathways in numerous cell types simultaneously [11], [12]. We previously employed this technique to stratify AML patients by signaling responses [13], map the complexities of the broader immune signaling network in primary murine cells [14], and automate the discovery of complex signaling relationships in primary human T cells using machine learning [15].

Several cytokines accelerate or slow SLE progression in both mouse and man and there is evidence that interferons play important roles in both species [2], [4], [6]–[8], [16]–[20]. The transcriptional signature of exposure to type I interferon is detectable in peripheral blood mononuclear cells (PBMC) of many SLE patients and nearly all juvenile SLE patients [7], [8], [21], [22]. Murine strains predisposed to developing lupus that are subsequently rendered homozygous for disruption of the IFNγ gene or treated with agents that deplete IFNγ have a longer life-span, a milder disease course, and far less renal damage than parental strains [17], [19] whereas infusions of exogenous IFNγ accelerated disease progression [23]. Similarly, knocking out the receptor for Type I interferons resulted in later disease onset, milder symptoms, and slower disease progression [16], [18]. A central role for cytokines in regulating SLE development is consistent with work in other systems showing that cytokines orchestrate the initiation, execution, perpetuation, and resolution of productive immune responses and play critical roles in coordinating interactions among numerous specialized cell types.

The most direct evidence that interferons have a causal role in human SLE comes from the high prevalence of SLE-associated symptoms among patients treated with type I interferons for conditions like hepatitis and cancer. In one study, 22% of patients treated with IFNα developed a positive blood test for ANA and 19% developed frank autoimmunity with one patient meeting the criteria for SLE diagnosis [24]. Interestingly, most patients' autoimmune symptoms were reversed when IFNα therapy was withdrawn. These findings show that high levels of IFNα can induce a form of autoimmunity, that this IFNα autoimmunity can be induced in a surprisingly large fraction of humans, and that maintenance of this autoimmunity requires consistently high levels of IFNα.

This and other research in mice and humans has led to a reevaluation of models in which autoimmunity is portrayed as rare and irreversible, held at bay by flawless tolerance. Central tolerance was shown to be less than perfect by the discovery that autoreactive T and B cells are a part of the peripheral repertoire of healthy individuals and that these cells can be activated when peripheral tolerance is overcome [25]. The frequency with which IFNα therapy induces autoimmunity is part of a body of evidence that tolerance can be overcome in a clinical context and raises the possibility that transient, interferon-induced autoimmunity might occur more often than previously thought. For example, IFNα levels can be very high during viral infections, notably higher than in many SLE patients. Thus, it might be that cytokine-driven activation of autoreactive lymphocytes is not unique to SLE patients; however, most individuals do not develop persistent autoimmunity due to intrinsic processes that inhibit the cytokine-driven feed-forward loops that maintain the immune state, thereby allowing the immune system to “stand down” once the pathogen is cleared.

Although the mechanisms involved in the initiation and early progression of SLE are thought to be key to understanding the disease, these processes occur prior to symptomatic manifestations that drive patients to seek medical assistance. A distinct advantage of murine models of SLE is that animals can be evaluated prior to disease onset and the animals also have relatively consistent disease progression. To test the hypothesis that cytokine signal transduction is modulated in both pathway- and cell-specific manners that correlate with SLE disease status, we analyzed samples collected before disease onset through initiation to the terminally severe phase using the MRL/*lpr* murine model of SLE. MRL mice spontaneously develop autoimmunity with many of the features of human SLE, including a similar autoantibody profile, autoimmune skin and liver disease, and immune-complex glomerulonephritis [9], [26], [27]. A spontaneous mutation, *lpr*, arose on the MRL background that greatly accelerated the autoimmune predisposition of MRL mice. In this report, MRL mice homozygous for the *lpr* mutation will be referred to as “*lpr*” and MRL mice homozygous wild type at this locus will be referred to simply as “MRL”. The onset and progression of SLE in female *lpr* mice is extremely consistent [28]. Autoantibody levels are detectable as early as 6 weeks and a pronounced lymphoadenopathy is observed at 12 weeks that is largely due to the proliferation of a population of B220+, TCRβ+, CD3+ cells that are mostly CD4− and CD8−. Multiple organs are affected and a steady deterioration of renal function manifests as heavy proteinuria beginning around 16 weeks of age. Very few *lpr* mice live past 28 weeks [28]. As such, this murine model is well suited for evaluation of processes that underlie each stage of SLE and provides a solid foundation for comparisons with other murine models of this disease as well as progression of the human disease.

Of the many signaling systems that are likely important to immune function, we chose to focus on cytokine stimuli and signaling pathways thought to have causal or secondary relationships to SLE and that are proximal to initiating events during immune activation. Inasmuch as others have used the production of cytokines as a baseline for how autoimmune processes guide immune cell output, we surmised that phosphorylation of STAT members in response to a panel of cytokines would provide an even more vital understanding of how immune cells change how they process immunomodulatory cues during SLE progression. We observed a strong correlation between SLE disease status and the ability of different subsets of primary immune cells to activate specific STAT signaling pathways in response to a broad range of cytokine stimuli. Notably, there was potent attenuation of IFNα, IFNγ, and IL-6 signaling across multiple immune cell subsets that was correlated to periods of increased SLE disease activity, suggesting that previously unrecognized, negative feedback loops oppose critical cytokine activities. As existing models of biological oscillators depend on the presence of negative feedback loops, often coupled with positive feedback loops [29], our identification of negative feedback regulation of IFNα signal transduction may provide a mechanistic explanation of why many SLE patients oscillate between flare and remission. Our identification of pathway specific and cell specific dysregulation may have significant implications for disease monitoring and treatment of SLE in human patients.

## Results

### Phospho-profiling of cells from MRL/lpr mice

In preparation for subsequent phospho-profiling experiments, we stained splenocytes from MRL and *lpr* mice at varying stages of disease (5–20 weeks) with antibodies specific to surface markers considered relevant to disease progression: B220, CD11b, Ly6C, Ly6G, Gr-1, CD19, IgM, CD21, CD3, TCRβ, TCRγδ, CD4, and CD8 [30], [31]. From this analysis (Fig. S1 and data not shown), we determined that use of B220, CD11b, TCRβ, CD4, and CD8 for phospho-specific flow cytometric analysis would capture most subsets important to disease progression and allow accurate resolution of populations that changed over time. All antibodies used for phospho-specific flow cytometric analysis were validated as previously described [14], [32].

**Figure 1:**
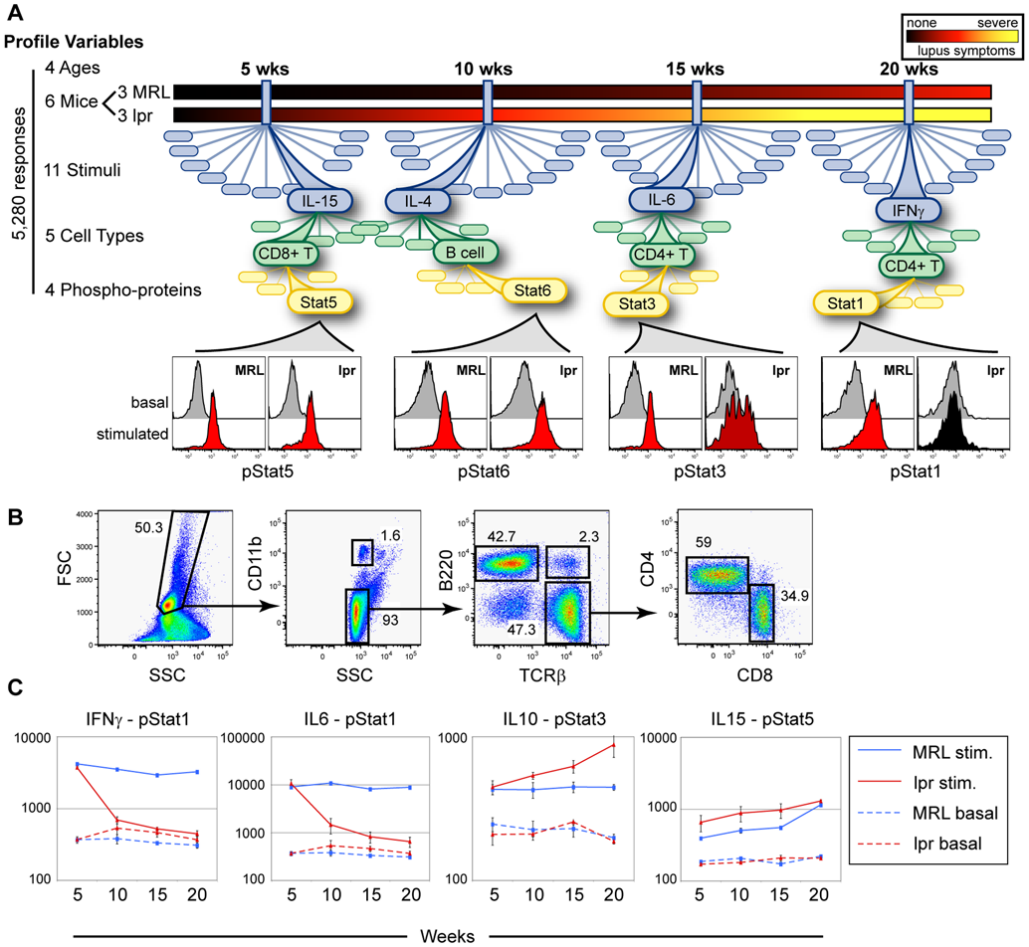
Experimental design for profiling changes in the murine immune signaling network during SLE progression. (A) MRL and *lpr* mice (n = 3 each) were profiled at four different ages (5, 10, 15, and 20 weeks). At 20 weeks of age, *lpr* mice show severe lupus symptoms, while MRL mice display only mild signs of disease. At each age, the phosphorylation of four Stat proteins (Stat1, Stat3, Stat5, and Stat6) in response to 11 different stimuli (basal, IL-2, IL-4, IL-6, IL-9, IL-10, IL-13, IL-15, IL-21, IFNα, and IFNγ) was analyzed in five cell types (B cells, CD11b-hi cells, CD4+ T cells, CD8+ T cells, and B220+TCRβ+ cells). Combination of these variables generated 5,280 response patterns. The histogram pairs under each age display basal and stimulated states for MRL and *lpr* mice for one signaling pathway in one particular cell type (e.g., IL-15 activation of Stat5 in CD8+ T cells at 5 weeks of age). The color of the stimulated histogram indicates fold change upon stimulation, black indicating no change, and red indicating >3 fold change. (B) Surface marker analysis used to define five cell types for phospho-flow analysis. Cells were size gated to exclude debris and red blood cells then gated for CD11b expression to yield CD11b-hi cells. Staining with B220 and TCRβ revealed three populations, B cells, T cells, and B220+TCRβ+ cells. The T cells were further sub-gated in CD4+ and CD8+. Numbers represent percentage of cells in each gate. (C) Stimulation with exogenous cytokines reveals differences in Stat signaling. The median fluorescent intensity (MFI) of phospho-specific staining in CD4+ T cells averaged across multiple mice is plotted against the age of the mice. Error bars show the standard deviation. The dotted lines display staining in the basal (unstimulated) state and the solid lines show the staining after stimulation with the indicated cytokine. The stimulation used in each chart is IFNγ, IL-6, IL-10, and IL-15 respectively.

To create an understanding of cell-type specific changes as SLE progresses, we profiled the cytokine response of multiple major immune cell subsets (Fig. 1A) in two genotypes: *lpr* as the disease model and MRL as control. Spleens were harvested from three mice per genotype at 5, 10, 15, and 20 weeks of age to examine four stages of SLE progression. Due to a lack of clinical manifestations of disease, 5 weeks is thought to be pre-disease, whereas at 10 weeks, *lpr* mice have detectable ANA and are thought to be in an early stage of SLE. At 15 weeks, manifestations of organ damage (e.g., proteinuria) are detectable in many animals and at 20 weeks the disease is terminally severe. The dissociated splenocytes from each mouse were stimulated *ex vivo* with ten cytokines previously implicated in SLE development, Th1/Th2 bias, or immune system regulation. Phosphorylation of Stat1, 3, 5, and 6 on activating residues was measured in five subsets of immune cells: CD11b^hi^ neutrophils and monocytes, B cells, CD4+ T cells, CD8+ T cells, and B220+TCRβ+ double positive T cells. (Fig. 1B) Stat2 and Stat4 phosphorylation was not measured due to their limited activation by most cytokines in our profile. This profile was carried out over a 15 week period to map changes in signaling that occurred as the mice transited from a pre-disease state, through early and intermediate stages, to the terminally severe phase of SLE. Examples of signaling responses that showed progressive changes over the time course are shown (Fig. 1C).

**Figure 2:**
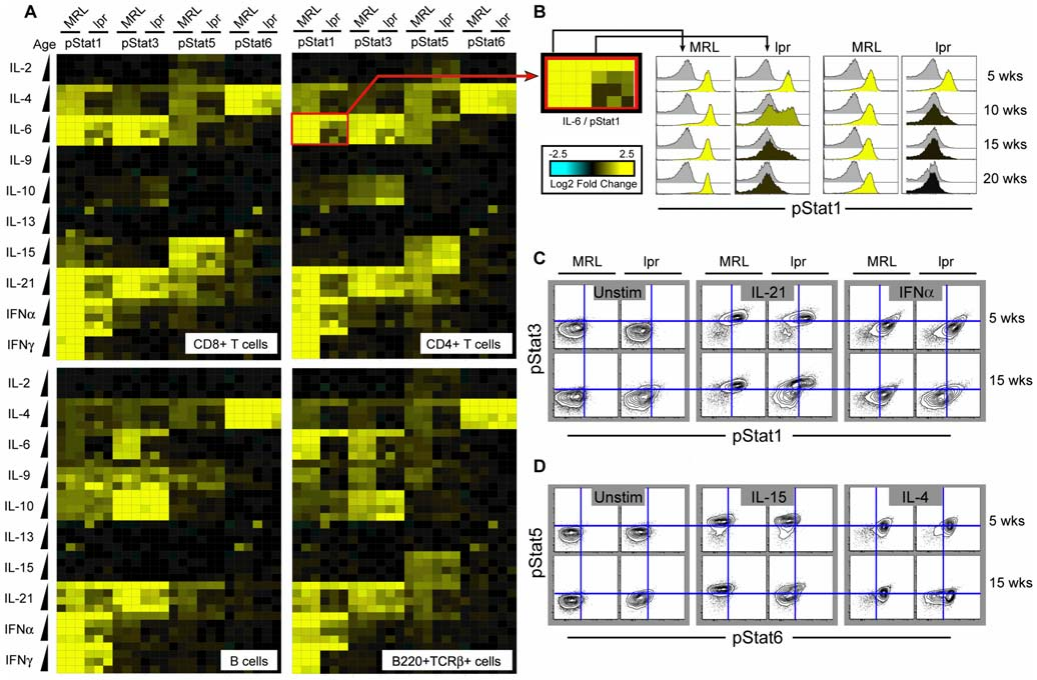
SLE progression is accompanied by reduced potentiation of multiple signaling nodes. (A) Heat map representation of Stat protein phosphorylation on activating residues induced by cytokine stimulation. Data is shown for CD4+ T cells, CD8+ T cells, B cells, and B220+TCRβ+ cells. Each square represents one signaling node which corresponds to the change in phosphorylation of one STAT protein in response to a cytokine in the specified cell type from one mouse. Each cytokine has four rows of data corresponding to stimulation of cells from mice at each age (increasing downward: 5, 10, 15, and 20 weeks). Three MRL and *lpr* mice were evaluated at each age; for a given row the first three columns of each Stat correspond to data from three MRL mice and the next three columns to that from three *lpr* mice. Note disease-associated changes in IFNα, IFNγ, IL-6 and IL-21 signaling. (B) Histograms show the phosphorylation of Stat1 in response to IL-6 (first two columns) or IFNγ (second two columns) in CD4+ T cells. The gray-shaded histograms are the pStat1 staining of the unstimulated cells and the color-shaded histograms are the staining of the samples stimulated with IL-6 or IFNγ. Note the bimodal histogram shape present in 10 week old *lpr* mice. (C) The entire dataset is multidimensional. Contour plots of Stat1 and Stat3 phosphorylation in CD4+ T cells in the unstimulated state, and in response to IL-21 and IFNα. (D) Stat5 and Stat6 phosphorylation in CD8+ T cells in the unstimulated state, and in response to IL-15 and IL-4.

The profile resulted in 5,280 nodes where each node is the phosphorylation status of one Stat protein in response to one cytokine (or in the unstimulated state) in a particular cell type (e.g., Stat3, IL-6, CD4+ T cells). Selected data is summarized in heat map format (Fig. 2A) with the cytokine-induced fold change in phosphorylation represented by yellow (increase) or blue (decrease). In the absence of exogenous stimulation there was no detectable activation of Stat3 or Stat5 over the disease course, though there was low but detectable increase in staining for pStat1 and pStat6 that was larger in B cells than in T cells (Fig. 1C, 2B, C, D and Fig. S2). The cytokine response, however, in four of the five cell types analyzed changed dramatically over the course of the disease.

**Figure 3:**
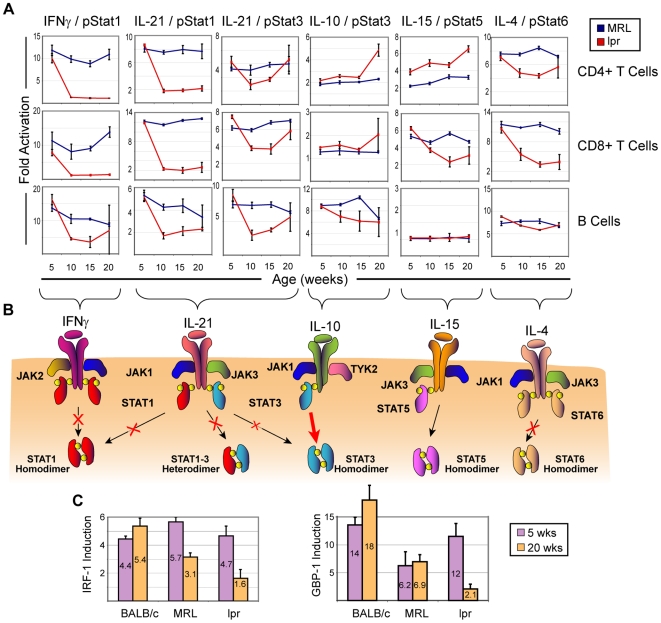
SLE progression is accompanied by highly specific blockade of cytokine-induced STAT signaling and target gene induction. (A) Detailed analysis of 24 signaling nodes. Data points are averaged from three mice; error bars represent standard deviation. By 10 weeks of age there were large decreases in the cytokine/STAT responses of multiple splenic cell types from *lpr* mice and these changes were pathway- and cell-type specific. Note that inhibition of some pathways (e.g., IL-4/pStat6) was cell-type specific. (B) Diagrams outlining the cytokine/STAT pathways modulated. (C) Real-time RT-PCR was used to quantify GBP-1 and IRF-1 transcript abundance in BALB/c, MRL, and *lpr* splenocytes taken from mice at 5 and 20 weeks of age in the absence and presence of IFNγ. Measurements were normalized to β-actin internal controls prior to calculation of fold induction.

Cytokine-induced signaling in cells from MRL and *lpr* mice was comparable at 5 weeks of age, prior to disease development, but differences became apparent at 10 weeks, when *lpr* mice began to show symptoms. In several cell types, there was a decrease in responsiveness to stimulation by certain cytokines (Fig. 2A). Interestingly, the loss of responsiveness to some cytokines was initially non-uniform: at 10 weeks of age the Stat1 response to IL-6 was ablated in roughly half of CD4+ T cells while it was high in the other half (Fig. 2B). CD4+ cells also showed heterogeneity in their Stat1 response to IFNγ as well (data not shown). There was also differential regulation of pathways activated by the same cytokine receptor. For example, in T cells from *lpr* mice there was a sharp drop in the Stat1 response to IL-21 but a comparatively modest reduction in the Stat3 response to this cytokine (Fig. 2C). This pathway specific regulation is even more pronounced when IL-6 is the stimulus (see below).Although differences between MRL and *lpr* mice were first seen at 10 weeks of age, the disparity in signaling responses became even greater at 15–20 weeks. Both genotypes showed a downward trend in the Stat1 response to IFNα as the mice aged, but the *lpr* mice showed an almost complete blockade of this pathway in all cell types starting at 10 weeks of age (Fig. 3A). A similar genotype-specific trend was also apparent for the Stat1 response to IFNγ and Stat6 response to IL-4; these responses remained relatively strong in MRL mice throughout the time course, but were greatly reduced in *lpr* mice from 10 weeks onward (Fig. 3A). Interestingly, while the Stat1 response to IFNγ was completely abolished from 10 weeks onward in all cell types, the Stat6 response to IL-4 remained comparatively strong in B cells, suggesting Stat1 and Stat6 are regulated by different mechanisms in this cell type.

Regulation was not limited to decreased signaling responses. T cells from *lpr* mice had an enhanced responsiveness to IL-10 at 20 weeks of age compared to the response at 5 weeks (Fig. 3A). T cells from pre-disease animals or wild type strains showed weak signaling responses to IL-10 and consistent with this, from five to fifteen weeks of age B220-TCRβ+ T cells of MRL and *lpr* mice had responses to IL-10 comparable to those of T cells from BALB/c mice (data not shown). At 20 weeks, however, the Stat3 response to IL-10 in B220- T cells from *lpr* mice roughly doubled compared to the response at 5 weeks and was far greater than that observed in T cells from MRL or BALB/c mice (Fig. 3A and data not shown). At every age, the B220+TCRβ+ T cells of both genotypes showed a much larger Stat3 response to IL-10 than single positive T cells. Elevated responses to IL-10 are of interest because this cytokine has been implicated in murine and human SLE in a number of studies [1]–[5].

The mechanistic changes in signaling derived from this dataset are organized by receptor in the diagram shown in Fig. 3B. Given the broad down-regulation of the Stat1 phosphorylation response to cytokines in *lpr* mice, we asked whether this lead to functional consequences in the cognate cells. The most immediate downstream implications of impaired activation of Stat1, a transcriptional regulator, should be reduced induction of target gene transcription by agents that signal through Stat1. Splenic suspensions from 5 and 20 week old BALB/c, MRL, and *lpr* mice were stimulated with IFNγ. The levels of mRNA expression of two representative Stat1 target genes, GBP-1 and IRF-1 [33], [34], were determined by a two-color real-time reverse transcriptase PCR (RT-PCR) assay. Consistent with the Stat1 signaling results, IFNγ stimulation strongly induced expression of both target transcripts in 5-week, but not 20-week, *lpr* splenocytes (Fig. 3C). Age did not impair induction of GBP-1 in MRL splenocytes or induction of either transcript in BALB/c splenocytes. Interestingly, significantly less IRF-1 was induced in MRL splenocytes at 20 weeks than at 5 weeks, though induction was stronger at both time points than in *lpr* mice. Although 20 week MRL mice had, as expected, greater Stat1 signaling and target gene induction than age-matched *lpr* mice, the Stat1 response to IFNγ was weaker in MRL splenocytes at 20 weeks than at 5 weeks and this reduction in signaling was enough to partially impair induction of IRF-1 expression. Thus, changes in signaling detected by phospho-specific flow cytometry correlated with relevant downstream transcription events in primary immune cells.

### Elevated SOCS1 protein levels correlate with differential inhibition of STAT activation during SLE progression

**Figure 4:**
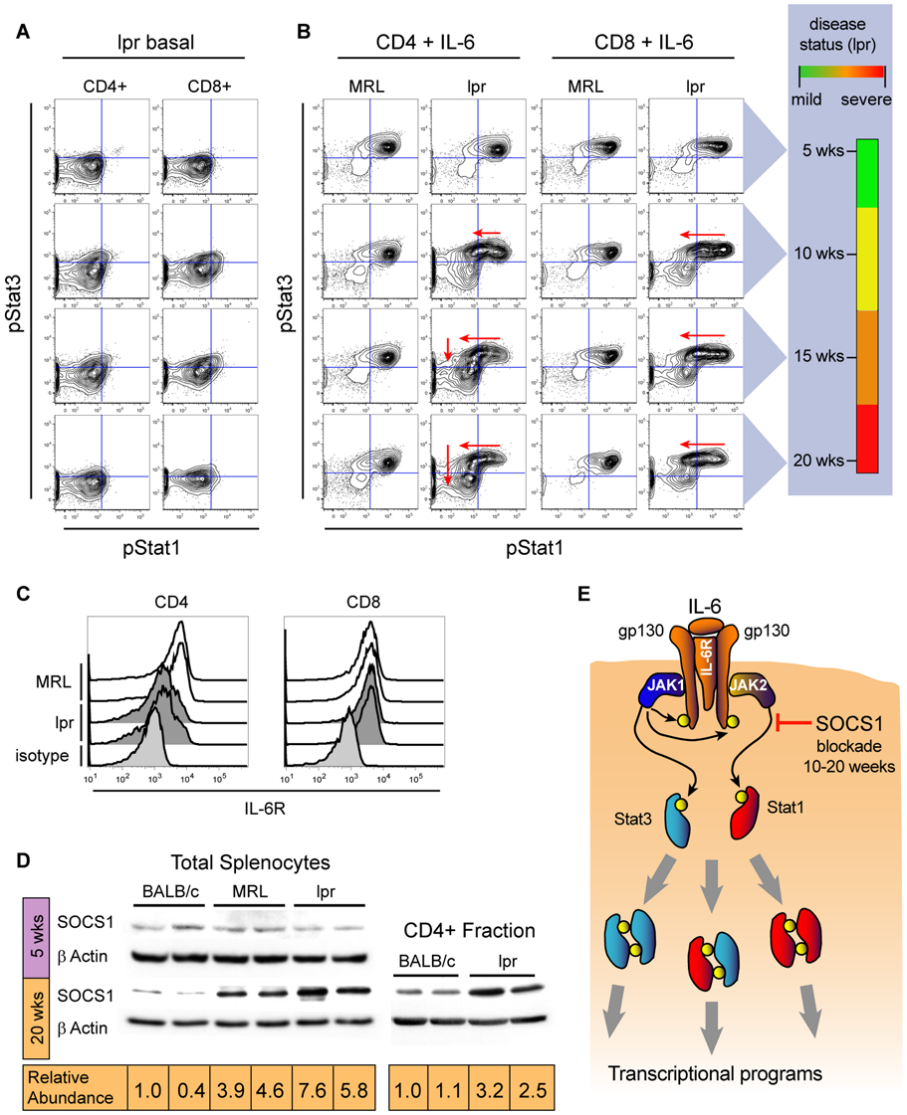
Differential inhibition of pStat1 and pStat3 signaling responses to IL-6 correlates with increased expression of SOCS1 protein. (A) Basal phosphorylation of Stat1 and Stat3 in B220-, CD4 and CD8 single positive T cells from *lpr* mice at 5, 10, 15, and 20 weeks of age. The same subsets from MRL mice have basal signaling that does not change with age and resembles that of 5-week-old *lpr* mice (not shown). (B) Phosphorylation of Stat1 and Stat3 in CD4 and CD8 single positive T cells after stimulation with IL-6. Nearly all single positive T cells from MRL mice show strong phosphorylation of Stat1 and Stat3 after stimulation with IL-6 and that did not change with age. In *lpr* mice, starting at 10 weeks, a large proportion of CD8+ T cells lost the pStat1 response to IL-6 but maintained pStat3 response to this cytokine at every age. Cells are in the upper left quadrant because, at the single-cell level, there is strong IL-6-induced phospho-activation of Stat3, but little or no detectable phospho-activation of Stat1. CD4+ T cells from 10-week-old *lpr* mice had no detectable pStat1 response to IL-6 and at later time points an increasing proportion of CD4+ T cells lost the pStat3 response as well. To the right of these two-dimensional plots is a set of color bars that summarize the severity of SLE in the *lpr* mice through time. (C) Flow cytometric analysis of IL-6 receptor expression on CD4 and CD8 single positive T cells at 20 weeks of age. (D) Western blot analysis of SOCS1 protein levels at 5 and 20 weeks in splenocytes from BALB/c, MRL, and *lpr* mice and from the CD4+ fraction. The relative abundance of SOCS1 at 20 weeks normalized to βActin is shown numerically under the blots. (E) Model of pathway-specific IL-6 signaling blockade. Differential blockade of pStat1 response to IL-6 may be due to selective inhibition of JAK2 activity by SOCS1. JAK2 activity is required for Stat1 phosphorylation but dispensable for Stat3 phosphorylation.

At the single-cell level, the Stat1 and Stat3 pathways were differentially regulated as SLE progressed. This was readily apparent for IL-6, a cytokine whose receptor is responsible for activating both Stat1 and Stat3 pathways in T cells. Basal activation of Stat1 and Stat3 in B220-CD4+ and B220-CD8+ T cells from *lpr* mice showed little detectable change throughout the course of the disease (Fig. 4A) and no change was see in T cells from age-matched MRL mice. In contrast, starting at 10 weeks of age a large fraction of the B220-CD8+ T cells from *lpr* mice had a poor pStat1 response to IL-6 despite the pStat3 response remaining strong (Fig. 4B column 4, compare row 1 to rows 2–4). Cell subset specific analysis shows that Stat3 is strongly phosphorylated after IL-6 stimulation even in B220-CD8+ T cells that have an undetectable pStat1 response. A similar effect was observed with B220-CD4+ T cells from 10 week old *lpr* mice (Fig. 4B); however, a large proportion of B220-CD4+ T cells from older *lpr* mice had poor pStat1 and pStat3 responses to IL-6 (Fig. 4B column 2, compare row 1 to rows 3 and 4). This inhibition of both pathways in B220-CD4+ T cells may be partly explained by reduced expression of the IL-6 receptor on the surface of CD4+ T cells from 20 week old *lpr* mice (Fig. 4C). In contrast, B220-CD8+ T cells from *lpr* mice showed no detectable drop in the expression of the IL-6 receptor consistent with maintenance of the pStat3 response to IL-6. Differential regulation of two pathways activated by the same receptor suggested the involvement of inhibitory cytoplasmic factors. This was further supported by the finding of the SLE-associated blockade of Stat1 phosphorylation downstream of other receptors (IL-21, IFNα and IFNγ) making it plausible that Stat1 activation through multiple receptors was being inhibited by a common cytoplasmic factor. Since IL6 receptor levels were maintained on B220-CD8+ cells and IFNGR1 and IFNGR2 showed no detectable change in expression on any of the cell types that showed reduced Stat1 responses to IFNγ (data not shown), the observed inhibition of Stat1 signaling must involve mechanisms other than receptor downregulation.

The suppressor of cytokine signaling-1 (SOCS1) protein is known to be a potent cytoplasmic inhibitor of Stat activation by interferons, IL-4 and IL-6 [35]–[39]; we observed that signaling through these cytokines was inhibited during SLE. Cells from SOCS1 knockout mice showed that, at physiological levels, SOCS1 has no effect on the magnitude or duration of IL6 induced Stat3 activation but does have a significant inhibitory effect on the magnitude of IL6 induced Stat1 activation [40], so it is possible that SOCS1 is responsible for the pathway-specific regulation of IL-6 signaling in early SLE. Therefore, we analyzed lysates from the splenocytes of BALB/c, MRL, and *lpr* mice for SOCS1 protein expression (Fig. 4D and Fig. S3). SOCS1 levels were low and nearly identical across the three genotypes at 5 weeks of age, but at 20 weeks there were significant differences between the genotypes and, as expected, SOCS1 protein expression inversely correlated with the ability of IFNγ to phosphorylate Stat1 and induce target gene transcription. The highest levels of SOCS1 were seen in 20 week *lpr* splenocytes; upon IFNγ stimulation these cells showed barely detectable increases in target gene expression. The elevated levels of SOCS1 protein were not simply due to increases in the B220+TCRβ+ compartment, as purified B220-TCRβ+CD4+ splenocytes from 20-week *lpr* mice showed high levels of SOCS1. In contrast, levels of SOCS3, another potent inhibitor of IL-6 signaling [39], [41], were not elevated in splenic lysates from 20 week MRL or *lpr* mice relative to those in age-matched BALB/c mice (Fig. S3).

## Discussion

There is a growing appreciation that inflammatory cytokines play key roles in the initiation and maintenance of autoimmune diseases such as SLE [42]. However, existing models do not account for the fact that cytokines thought to drive autoimmune disease are found at their highest levels not in autoimmune patients but in patients mounting protective immune responses to pathogens [42], [43]. Thus, the mere presence of an inflammatory state is not sufficient to drive autoimmunity, but aberrant control of immune cell responses to inflammatory cytokines may disrupt the delicate balance between immunity and self-reactivity. In this study, we observed dysregulation of cytokine signal transduction that correlated with disease activity in a mouse model of SLE. In healthy individuals, negative feedback regulation may be required to oppose the inflammatory state and restore homeostasis, but in SLE, regulation may be deficient and a chronic state of aberrant immune activation may result.

A significant finding of this study was several instances of pathways activated by the same receptor that were differentially regulated at the single-cell level. After the onset of SLE, the Stat1 response to IL-6 and IL-21 was poor in the same T cells that exhibited strong activation of Stat3 (Fig. 2C and Fig. 4B). This suggests that the observed differential regulation occurs in the cytoplasm, since reduced receptor expression would be expected to inhibit both pathways. We observed an increase in SOCS1 protein levels during SLE progression, suggesting that SOCS1 is a cytoplasmic mediator that plays a role in this pathway-specific regulation (Fig. 4C–E). Although over-expression studies have shown that SOCS family members are highly promiscuous in their inhibitory activities against JAK proteins, there is growing evidence that at physiological levels SOCS are more specific to particular STAT protein isoforms[40]. SOCS1 was first identified for its potent ability to inhibit JAK2 activity[35] and the mechanism for its JAK2 preference over JAK1 was later defined [44]. Knockout studies have shown JAK2 to critical for the Stat1 response to IL-6, but dispensable for the Stat3 response to this cytokine[45]. The selective inhibition of the Stat1 response to IL-6 we observed in early SLE could be due in part to moderate levels of SOCS1 that specifically target JAK2 activity while having relatively little influence on JAK1 (Fig. 4E). This suggests a model in which SOCS1 levels rise during SLE and inhibit the Stat1 responses to IL-6, IL-21, and interferons with comparatively little influence on Stat3 signaling including the Stat3 responses to IL-6 and IL-10. Consequently, one would expect that genes whose promoters are driven by Stat1 homodimers (or Stat1-Stat3 heterodimers) would be inhibited in their normal regulatory patterns starting from early SLE onset. This provides direct mechanistic consequences for immune activities driven by these transcription factors.

The MRL/*lpr* model is only one of several models of SLE, all of which are understood to be imperfect representations of human disease [46]. However, *lpr* mice do exhibit many of the features of human SLE, including a similar autoantibody profile, autoimmune skin and liver disease, and immune-complex glomerulonephritis. As with other murine models of SLE, interventions that oppose interferon action reduce the severity of the disease in these mice. In this study, we observed strong negative regulation of signaling responses to cytokines thought to have causal roles in murine and human SLE. Importantly, we found that many of the signaling responses that were ablated during the progression of SLE in the *lpr* mice were also reduced in cells from SLE patients with non-zero SLE Disease Activity Index (SLEDAI) scores (in preparation).

Given the presumed role of interferon in SLE, it was surprising that the phosphorylation of Stat1 and the transcriptional response to interferons was greatly reduced as SLE progressed in *lpr* mice. Although this seems counterintuitive, both Type I and Type II interferons have been shown to potently induce negative feedback regulation of Stat1, in part by inducing SOCS1 [37]–[39], [47], [48]. SOCS1 levels were elevated in the *lpr* mice (Fig. 4D) and in SLE patients (in preparation). Indeed, a blockade of Stat1 signaling may be the inevitable result of interferon activity and this blockade has been shown to be induced during protective Th1 responses, potentially allowing lymphocytes to survive the deleterious effects of prolonged interferon exposure [49]. Since infection is a leading cause of death for SLE patients [50], it is possible that a prolonged state of reduced responsiveness to IFNα may further compromise a patient's ability to fight infection. The blockade of Stat1 signal transduction and elevated SOCS1 protein levels we observed in the progression of murine SLE suggest that between 5 and 10 weeks-of-age, strong interferon signaling induces negative feedback regulation of Stat1. Consistent with this, Stat1 basal activation was detectably elevated at 10 weeks-of-age even though exogenous interferon stimulation was not able to induce the high level of Stat1 activation observed in age-matched MRL mice. From 10 weeks onward in *lpr* mice, Stat1 activation in response to exogenous stimulation was greatly reduced compared to MRL mice, but IFNγ was still able to induce some expression of target genes GBP-1 and IRF-1, though induction was markedly weaker than when Stat1 signal transduction was strong (Fig. 3C).

In the context of SLE, this negative feedback regulation of cytokine signal transduction fails to permanently silence autoimmunity and instead may play a role in destabilizing the immune system. Biological oscillators have been found to require negative feedback [29]. Different relative contributions of negative and positive feedback result in a bi-stable system, a system that is essentially “on” or “off” but has no stable intermediate state [51]. An effective immune system is designed to be bi-stable: fully activated in response to pathogen challenge and quickly turning completely off when the pathogen is cleared. It is possible that disturbances in the coupling of negative and positive feedback elements, such as interferon signaling, may destabilize and shift the immune system from a bi-stable system to one that oscillates between flare and remission as observed in many SLE patients. Consistent with this hypothesis, we have found negative regulation of the same cytokine signaling pathways reported here is present in a large fraction of SLE patients, and robust changes in cytokine signaling were found to precede flare (manuscript in preparation). Oscillations between flare and remission are not observed in all human patients, or in murine models, therefore a longitudinal study of patients transiting between flare and remission will be required to better understand the role of feedback regulation. Our identification of negative feedback regulation of interferon signaling in this study provides evidence that an interferon-driven oscillator could play a role in cycling between flare and remission.

In summary, we find that changes in STAT signaling parallel SLE progression in a murine model of SLE. Critically, basal phosphorylation alone did not robustly discriminate disease states from each other or from non-diseased individuals, but interrogation of cells with environmental cues (cytokines) revealed altered signaling nodes that did correlate with disease progression. This is consistent with our observations of human leukemia [13] where basal signaling was of limited value in predicting clinical outcome, but signaling *responses* were indicative of cell character and were of significant prognostic value. Thus, in conjunction with conventional indicators, quantifying the disturbed nature of cytokine action in SLE could lead to approaches that objectively characterize disease status at the level of single-cell signaling states and may point to therapies that safely modulate the disease by modifying cell and cytokine responses.

## Materials and Methods

### Antibodies and reagents

Antibodies against murine CD3 (clone 145-2C11), TCRγδ (GL-3), TCRβ (H57-597), CD4 (GK1.5), CD8 (53–6.7), B220 (RA3-6B2), CD19 (ID3), CD21 (7G6), IgM (11/41), Ly6C (AL-21), Ly6G (1A8), Gr-1 (RB6-8C5), and CD11b (M1/70) were kindly provided by BD Biosciences (La Jolla, CA). Surface antibodies were conjugated to Alexa 488, FITC, PE, Cy5.5.PerCP, Cy7PE, Pacific Blue, APC, Alexa 700, or Cy7APC. Antibodies against the phospho-proteins Stat1 Y701 (4A), Stat3 Y705 (4), Stat5 Y694 (47), Stat6 Y641 (J71-773.58.11) conjugated to Alexa 488 or Alexa 647 were also provided by BD Biosciences. SOCS-1 antibody was from Zymed (clone 38–5200). Recombinant murine (rm) IFNγ, IL-2, IL-4, IL-6, IL-9, IL-10, IL-13, and IL-21 were from BD. rmIFNα was from R&D Systems (Minneapolis, MN). rmIL-15 was from Peprotech (Rocky Hill, NJ). All cytokines were used at a final concentration of 50 ng/mL, except for murine IL-10 which was used at 100 ng/mL and murine IFNα which was used at 100 U/mL. Dose-response experiments verified that these concentrations were at least 5-fold higher than the minimum concentration needed to achieve maximal phospho-activation of the downstream Stat. Taqman probes specific to murine β-actin, Gbp-1, and IRF-1 were obtained from Applied Biosystems (Foster City, CA). The one-step rtPCR enzyme mix used was Superscript with Platinum Taq (Invitrogen, Carlsbad, CA).

### Murine SLE sample preparation

MRL +/+ (MRL) and MRL *lpr*/*lpr (lpr)* mice were obtained from Jackson Laboratories (Bar Harbor, ME) and were housed at the Stanford Animal Facility from 5–20 weeks of age. All mice were handled in accordance with APLAC and Stanford University animal care guidelines. BALB/c mice were obtained from Jackson or from the in-house colony at Stanford and were age-matched to MRL and *lpr* mice. At the desired age, spleens were harvested and dissociated into a single-cell suspension at a concentration of 5×10^6^ cells/mL in RPMI-1640 containing 10% FBS and PSQ (RPMI-10).

### Murine surface marker analysis

Splenocytes were pelleted and resuspended at 10^7^ cells/mL in staining medium (PBS containing 0.5% BSA and 0.02% sodium azide). Appropriate surface marker antibodies were added at optimal dilutions and samples were incubated for 30 minutes on ice. Cells were then washed once with at least 20 volumes of staining media and analyzed by flow cytometry on a FACSCalibur instrument fitted with 488 and 633 lasers (BD Biosciences, San Jose, CA). At least 100,000 cells were analyzed for each sample.

### Cytokine stimulation and phospho-flow

Murine splenocytes were cultured at 37°C for 2 hours in RPMI-10 to allow for recovery from the splenic dissociation. Cytokines were added to the culture media and the cells incubated for 15 minutes at 37°C, fixed for 10 minutes at room temperature with formaldehyde directly added to the medium (1.5% final concentration), and then pelleted. The cells were resuspended in MeOH previously chilled to 4°C and then stored at −80°C until phospho-flow analysis. Samples were removed from −80°C storage, washed two times with staining media, and resuspended at 10^7^ cells/mL in staining media. Staining was with a cocktail of antibodies, including CD11b Ax405, TCRβ PE, B220 Cy5.5PerCP, CD8 Cy7PE, CD4 Cy7APC and the combination of pStat3 Ax488 and pStat1 Ax647 or the combination of pStat5 Ax488 and pStat6 Ax647. Samples were stained for 30 minutes, washed once with staining media, and analyzed on an LSRII flow cytometer equipped with 405, 488, and 633 nm lasers. Digital data was acquired with BD Diva software with >75,000 size-gated cells collected per data point. Data was analyzed in FlowJo software. Median fluorescence intensity (MFI) was used to calculate the fold change between stimulated and unstimulated cells: fold change = MFI_stim_/MFI_unstim_. A log_2_ fold change was derived so that samples showing no stimulation had a value of zero, those that showed increases in phospho-protein levels were positive, and those that showed decreases were negative. Flow cytometric data from this study can be found online at: http://proteomics.stanford.edu/nolan/.

### Real-time RT-PCR and western blotting

RNA preparations and lysates for western analysis were obtained from a fraction of each spleen immediately after harvesting to determine basal expression levels. The remaining cells were cultured as a single cell suspension at 37°C for 2 hours in RPMI-10. The suspensions were then cultured for an additional 2.5 hours in the presence or absence of IFNγ. The cells were pelleted and then lysed in Trizol for 15 minutes at room temperature. Samples were stored at −80°C in Trizol prior to RNA isolation. After extraction from Trizol and isopropanol precipitation, the RNA was further purified using RNeasy columns (Qiagen, Valencia, CA) and was resuspended in water. Transcript quantitation was normalized to β-actin internal controls. Fold induction was calculated as the ratio of transcript abundance after culture in the presence of IFNγ to abundance after culture in the absence of IFNγ. All western blots were performed with whole cell lysates, resolved by SDS-PAGE, transferred to PVDF (Immobilon-P, Millipore, Billerica, MA), and blotted for SOCS-1. Blots were quantified using ImageJ software and were analyzed in parallel using LumiAnalyst3.0 software. As a positive control, 293T cells were transfected with a SOCS1 expression construct in a pcDNA3.1 backbone and lysates were prepared after 24 or 36 hours.

## Supporting Information

Figure S1 Changes in splenic immune compartments during SLE. Splenocytes from MRL and *lpr* mice of the specified ages were stained with cocktails of surface antibodies (TCRβ, B220, CD4, CD8, CD19, CD21, IgM) and analyzed by flow cytometry. (A) Total splenocytes analyzed for B220 and TCRβ expression were gated into B cells (B220+TCRβ-), T cells (TCRβ+B220-), and B220+TCRβ+ cells. Note increases in B220+TCRβ population in *lpr* mice at 10–20 weeks. Other notable changes are indicated by highlights or arrows. (B) B cells (CD19+B220+) were analyzed for IgM and CD21 expression. Immature (CD21- IgM-hi), mature (CD21-int IgM-int), marginal zone (CD21-hi IgM-hi), and CD21-IgM- populations were gated. (C) T cells (B220-TCRβ+) were analyzed for CD4 and CD8 expression. (D) B220+TCRβ cells were also analyzed for CD4 and CD8 expression. Note the presence of a large double-negative population, characteristic of the *lpr* model.
(9.38 MB TIF)

Figure S2 SLE progression induces small differences in basal phospho-Stat1 and phospho-Stat6 staining. Splenic suspensions were not stimulated prior to fixation, permeabilization, and analysis. Shown is the phospho-specific staining of B220-TCRβ+CD4+ T cells and B220+TCRβ- B cells. Each point represents the median fluorescent intensity averaged across three mice and normalized to the median fluorescent intensity averaged across three 5 week old MRL mice. The error bars display the normalized standard deviation.
(0.09 MB TIF)

Figure S3 SOCS1 antibody validation and blotting SOCS1 and SOCS3. (A) The human fibroblast line 293T was mock transfected or transfected with a SOCS1 expression construct employing the pcDNA3.1 backbone. Lysates were prepared 24 and 40 hours after transfection, resolved by SDS-PAGE, transferred to PVDF membrane, and blotted with different anti-SOCS1 primary antibodies and then with appropriate HRP-conjugated secondary antibodies. The membrane shown here was blotted with Zymed 38–5200 as the primary. This antibody was used for all SOCS1 expression analysis of SLE material; it correctly recognized a 30 kDa band that was much more intense (>10 fold) in lanes containing lysates from cells transfected with the SOCS1 expression construct than those from control 293T lysates. In contrast, blots using Santa Cruz SC-7001 as primary did not show differential staining in the absence and presence of the SOCS1 expression construct so this antibody was not used in our analysis of SLE material. (B) Comparison of 20 week old BALB/c, MRL, and *lpr* splenic lysates showed differential staining with Zymed 38–5200 primary. This is an experimental replicate that shows the same trend displayed in Figure 4D. Quantitation of the SOCS1 bands normalized to <lower case beta>Actin is shown in the red bar graph below the blot. (C) MRL and lpr mice were found to have comparable levels of SOCS3 when analyzed using Santa Cruz SC-7009 as primary. Quantitation of the SOCS3 bands normalized to <lower case beta>Actin is shown in the blue bar graph below the blot.
(0.79 MB TIF)
