# Supplementary figures and images for: Stage Dependent Aberrant Regulation of Cytokine-STAT Signaling in Murine Systemic Lupus Erythematosus
Source: PLoS One. 2009 Aug 25;4(8):e6756. doi: 10.1371/journal.pone.0006756 (PMC2727051; doi:10.1371/journal.pone.0006756)

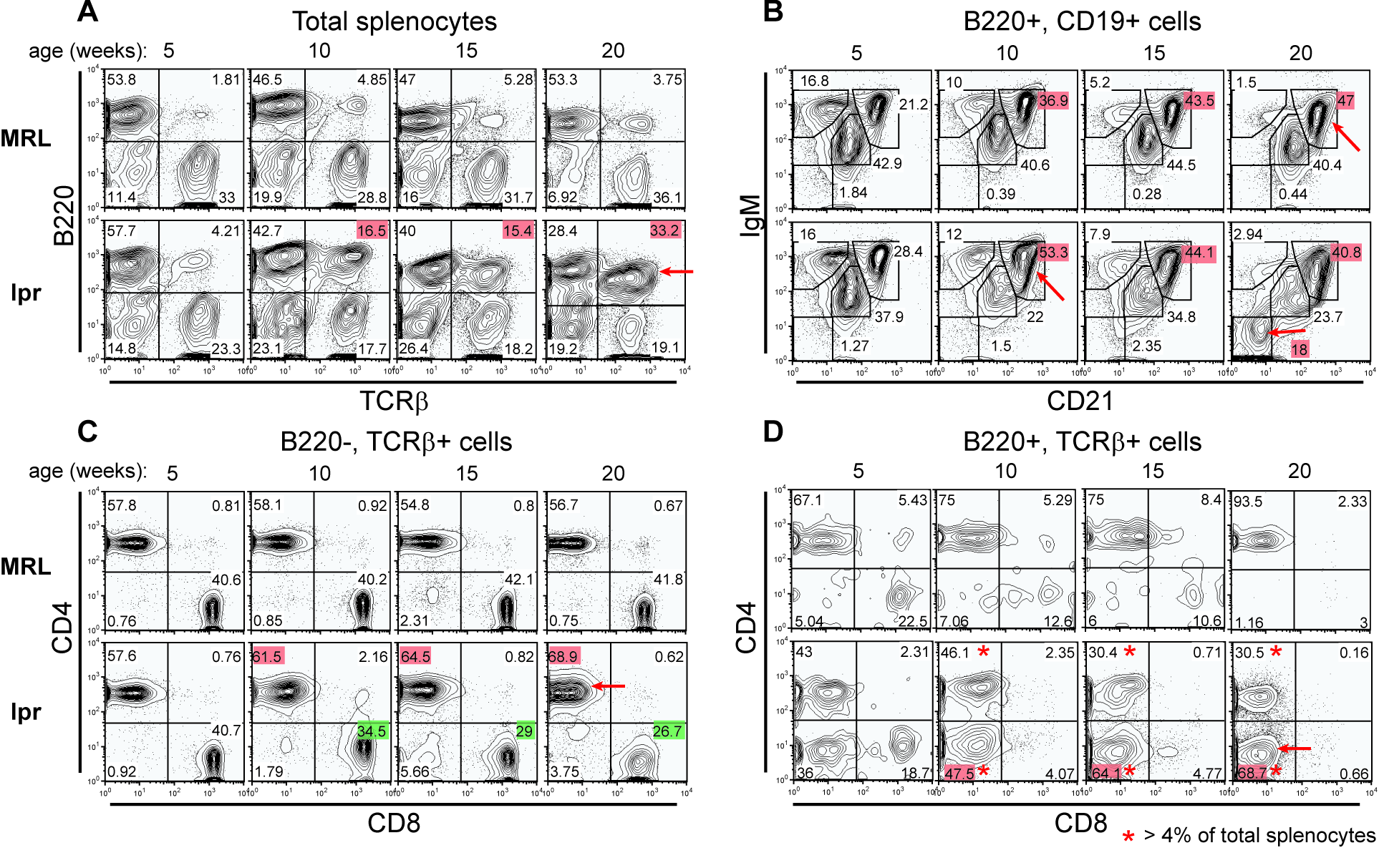

Supplement: Figure S1 — Changes in splenic immune compartments during SLE. Splenocytes from MRL and lpr mice of the specified ages were stained with cocktails of surface antibodies (TCRβ, B220, CD4, CD8, CD19, CD21, IgM) and analyzed by flow cytometry. (A) Total splenocytes analyzed for B220 and TCRβ expression were gated into B cells (B220+TCRβ-), T cells (TCRβ+B220-), and B220+TCRβ+ cells. Note increases in B220+TCRβ population in lpr mice at 10–20 weeks. Other notable changes are indicated by highlights or arrows. (B) B cells (CD19+B220+) were analyzed for IgM and CD21 expression. Immature (CD21- IgM-hi), mature (CD21-int IgM-int), marginal zone (CD21-hi IgM-hi), and CD21-IgM- populations were gated. (C) T cells (B220-TCRβ+) were analyzed for CD4 and CD8 expression. (D) B220+TCRβ cells were also analyzed for CD4 and CD8 expression. Note the presence of a large double-negative population, characteristic of the lpr model. (9.38 MB TIF) [file pone.0006756.s001.tif]

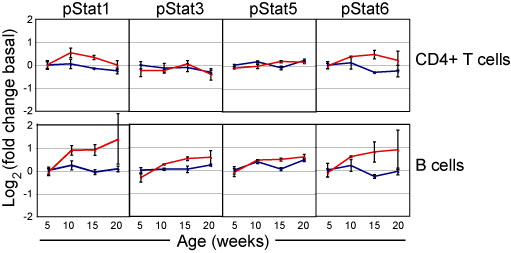

Supplement: Figure S2 — SLE progression induces small differences in basal phospho-Stat1 and phospho-Stat6 staining. Splenic suspensions were not stimulated prior to fixation, permeabilization, and analysis. Shown is the phospho-specific staining of B220-TCRβ+CD4+ T cells and B220+TCRβ- B cells. Each point represents the median fluorescent intensity averaged across three mice and normalized to the median fluorescent intensity averaged across three 5 week old MRL mice. The error bars display the normalized standard deviation. (0.09 MB TIF) [file pone.0006756.s002.tif]

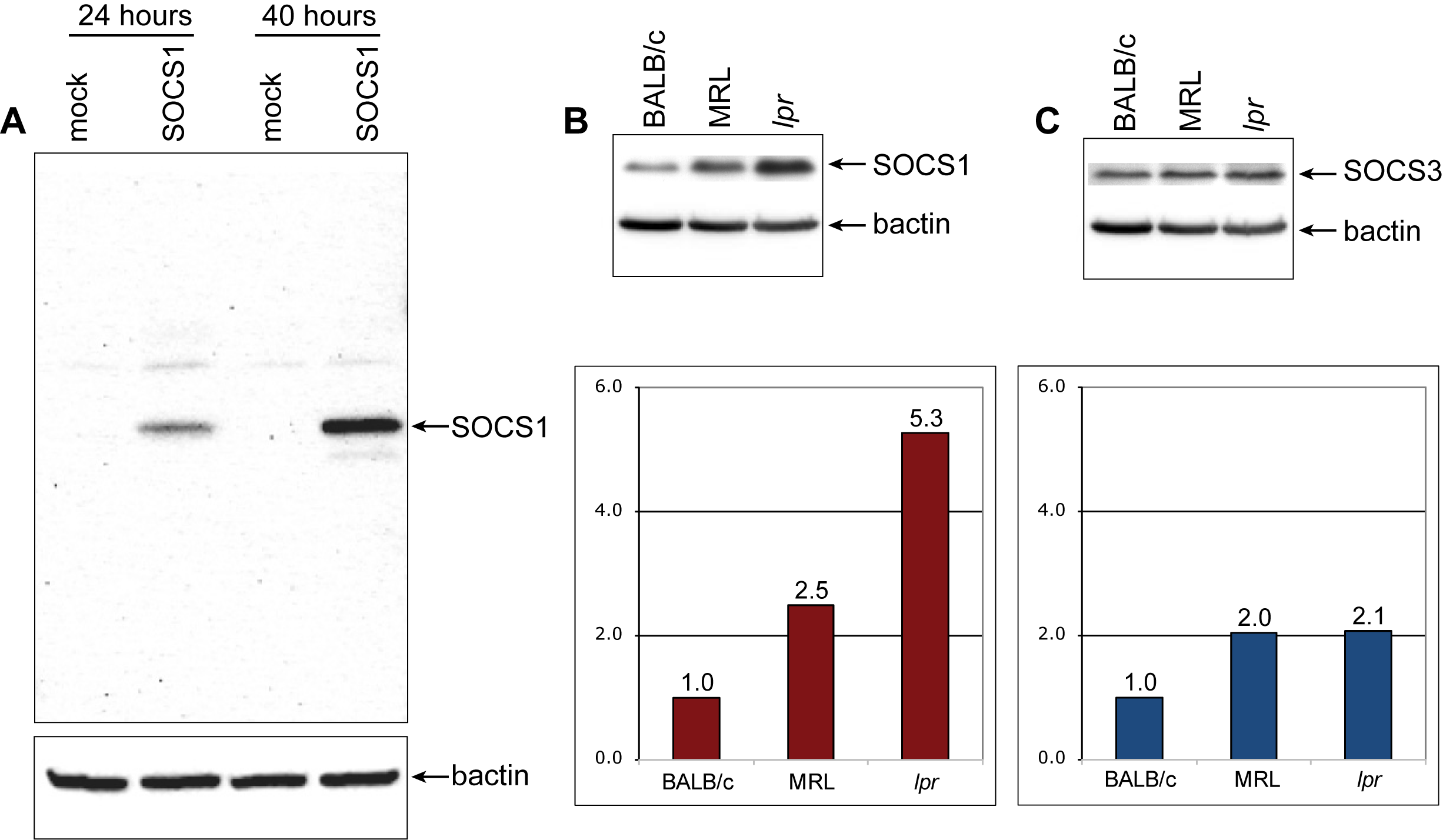

Supplement: Figure S3 — SOCS1 antibody validation and blotting SOCS1 and SOCS3. (A) The human fibroblast line 293T was mock transfected or transfected with a SOCS1 expression construct employing the pcDNA3.1 backbone. Lysates were prepared 24 and 40 hours after transfection, resolved by SDS-PAGE, transferred to PVDF membrane, and blotted with different anti-SOCS1 primary antibodies and then with appropriate HRP-conjugated secondary antibodies. The membrane shown here was blotted with Zymed 38–5200 as the primary. This antibody was used for all SOCS1 expression analysis of SLE material; it correctly recognized a 30 kDa band that was much more intense (>10 fold) in lanes containing lysates from cells transfected with the SOCS1 expression construct than those from control 293T lysates. In contrast, blots using Santa Cruz SC-7001 as primary did not show differential staining in the absence and presence of the SOCS1 expression construct so this antibody was not used in our analysis of SLE material. (B) Comparison of 20 week old BALB/c, MRL, and lpr splenic lysates showed differential staining with Zymed 38–5200 primary. This is an experimental replicate that shows the same trend displayed in Figure 4D. Quantitation of the SOCS1 bands normalized to Actin is shown in the red bar graph below the blot. (C) MRL and lpr mice were found to have comparable levels of SOCS3 when analyzed using Santa Cruz SC-7009 as primary. Quantitation of the SOCS3 bands normalized to Actin is shown in the blue bar graph below the blot. (0.79 MB TIF) [file pone.0006756.s003.tif]
